# Relationship between piglets’ survivability and farrowing kinetics in hyper-prolific sows

**DOI:** 10.1186/s40813-023-00332-y

**Published:** 2023-08-28

**Authors:** Alexandra Schoos, Bruno Bracco Donatelli Muro, Rafaella Fernandes Carnevale, Ilias Chantziaras, Evelien Biebaut, Geert Paul Jules Janssens, Dominiek Maes

**Affiliations:** 1 Department of Internal Medicine, Reproduction and Population Medicine, Faculty of Veterinary Medicine, Ghent University, Merelbeke, Belgium; 2 Department of Nutrition and Animal Production, School of Veterinary Medicine and Animal Sciences, University of São Paulo (USP), Campus Pirassununga, São Paulo, SP Brazil; 3 Department of Veterinary and Biosciences, Faculty of Veterinary Medicine, Ghent University, Merelbeke, Belgium

**Keywords:** Hyperprolific sow, Survivability, Colostrum, Mortality, Inter-piglet birth interval

## Abstract

**Background:**

Prolonged farrowing and more piglets born with low birth weight are undesirable consequences of genetic selection for increased litter size. The objective of the present observational study was to evaluate the relationship between piglets’ survivability and farrowing kinetics in hyperprolific sows. A total of 58 sows of different parities and 1190 piglets were included. The entire farrowing process was monitored and the following parameters were recorded: inter-piglet birth interval, birth order, total born, live born, dead born, and mummified piglets, obstetric intervention, weight at birth and 24h, colostrum yield and intake.

**Results:**

The sows included in this study had on average 20.6 ± 0.6 total piglets born, of which 16.4 ± 0.6 were live born, 3.3 ± 0.4 were stillborn and 0.9 ± 0.2 were mummified piglets. The average farrowing duration and average birth interval were 411.3 ± 31.6 and 20.6 ± 1.7 min, respectively. Farrowing duration was positively associated (*p* < 0.05) with parity, number of stillborn and mummified piglets. Piglet mortality 24h after birth was negatively affected (*p* < 0.01) by birth weight and positively affected (*p* < 0.01) by cumulative birth interval. The last tercile of piglets born (birth order ≥ 17) had the highest (*p* < 0.01) inter-piglet birth interval (IPBI) (43.4 ± 4.17 min) compared to piglets born in the first (birth order between 2 and 7) (26.5 ± 3.8 min) and second (birth order between 8 and 16) terciles (21.9 ± 3.8 min). Cumulative birth interval, birth weight, occurrence of stillborn piglets and manual intervention were positively associated (*p* < 0.05) with IPBI. Piglet birth weight was also positively associated (*p* < 0.01) to individual colostrum intake. Piglets ingesting more colostrum had lower (*p* < 0.01) mortality from 24h after birth until weaning. Sow’s parity and cumulative birth interval were positively associated with the presence of stillborn piglets (*p* = 0.02 and *p* < 0.01, respectively).

**Conclusion:**

Reducing farrowing duration may be crucial to decrease stillbirth rate and neonatal mortality in hyperprolific sows. Moreover, special care must be provided to the lighter piglets within a litter to increase their colostrum intake and minimize piglet’s mortality throughout lactation.

**Supplementary Information:**

The online version contains supplementary material available at 10.1186/s40813-023-00332-y.

## Background

Prolonged farrowing (> 300 min) and high inter-piglet birth interval (IPBI) (> 20 min) are arguably the most important factors leading to both increased stillbirth rate and increased preweaning mortality observed in modern hyperprolific sows i.e. sows giving birth to more than 16 piglets, compared to older genotypes [1–3]. The precise impact of these factors on piglets’ survivability remains to be further elucidated and quantified. Some studies defined dystocia based on IPBI and suggested that this variable is determinant to stillbirth occurrence [4]. Others argue that the interval from onset of farrowing until the birth of a given piglet is rather more important than individual IPBI due to the cumulative occlusion of umbilical cord that leads to greater risk of damage or rupture of the umbilical cord in such a manner that asphyxia arises [5].

Piglets with greater birth order show more signs of asphyxia such as higher lactate concentration and lower blood pH compared to piglets born in the beginning of farrowing [6–8]. Consequently, the probability of delivering a stillborn or a piglet with compromised vitality increases as the birth order increases [8, 9]. Thus, it is questionable whether IPBI contributes to stillbirth as much it has been suggested [8].

The positive association between farrowing duration and stillbirth rate is however more consistent. According to Tummaruk et al. [10], sows with prolonged farrowing (> 240 min) had a 3.5-fold higher stillbirth rate than sows with short farrowing (< 120 min). Similarly, Udomchanya et al. [11] showed that sows that do not give birth to stillborn piglets have a 152 min shorter farrowing process than sows that gave birth to three or more stillborn piglets. Farrowing duration has also been linked with fertility as a longer farrowing process was associated with a higher repeat breeding rate after weaning [12]. Environmental factors play a key role in the progress of farrowing in pigs. Sows with access to nest-building material as well as sows housed in free farrowing systems seem to have fewer complications during and after farrowing such as prolonged farrowing duration, high stillborn rate, and delayed uterine involution [13–15]. The mechanisms leading to increased farrowing duration need to be further explored as several factors related to sows, piglets, environment and peripartum management might be involved [4, 16–18].

Prolonged farrowing might not only lead to an increased stillbirth rate, it may also negatively affect the vitality, colostrum intake, growth, and survivability throughout lactation [2, 7]. Perinatal mortality remains an unsolved problem in swine operations, accounting for 50–80% of overall piglet mortality [19]. Crushing has proven to be the main cause of perinatal death in swine operations but, as some studies have demonstrated, it is often only the last event in a series of previous causal effects (hypothermia, starvation, diarrhea) that frequently result from complications that sows and piglets experienced during farrowing [20, 21].

Elucidating the variables interconnecting farrowing kinetics and piglets’ survivability is an essential first step to minimize perinatal losses. Therefore, the aim of the present observational study was to investigate the relationship between piglets’ survivability and farrowing kinetics in hyperprolific sows.

## Results

All descriptive data of the sows, the farrowing process and the litter performance are shown in Table 1.

**Table 1:** Descriptive data of the sows, the farrowing process, and the litter performance

Variable	Mean ± SE	Min	Max	Observations
Parity	3.3 ± 0.3	1	9	58
Gestation length (days)	115.8 ± 0.1	115	117	58
Sow’s rectal temperature 24h after farrowing	38.0 ± 0.1	37.5	39.5	58
Subsequent weaning to estrus interval (days)	3.8 ± 0.1	1	5	53
Total born (n)	20.6 ± 0.6	9	31	58
Live born (n)	16.4 ± 0.6	5	24	58
Stillborn (n)	3.3 ± 0.4	0	16	58
Stillborn (%)	15.4 ± 1.7	0	64	58
Mummified piglets (n)	0.9 ± 0.2	0	9	58
Mummified piglets (%)	4.2 ± 0.9	0	42	58
Farrowing duration (min)	411.3 ± 31.6	128	1222	58
Average birth interval (min)	20.6 ± 1.7	6	87	58
Piglet’s mortality 24h after birth (%)	5.3 ± 1.0	26	0	58
Piglet mortality between 24h post-farrowing and weaning (%)	20.6 ± 1.6	46	0	58
Backfat entrance (mm)	15.6 ± 0.5	8	27	58
Backfat farrowing (mm)	14.7 ± 0.4	9	24	58
Backfat weaning (mm)	12.8 ± 0.4	8	22.5	57
Average litter daily gain until weaning (kg)	2.13 ± 0.1	0.7	3.1	58
Colostrum yield (g)	4558 ± 168	7578	725	58

All the results obtained from univariable or multivariable regression models are summarized in an overview table (Table 2).

**Table 2:** Summary of the results obtained from univariable or multivariable regression models

Dependent variable	Independent variable	*p*-value
Piglet level		
Inter-piglet birth interval (min)	↑ Cumulative birth interval (min)	< 0.01
	↑ Piglet’s birth weight (kg)	0.03
	↑Stillborn piglets (n)	< 0.01
	↑Manual Intervention (yes/no)	< 0.01
Colostrum intake (g)	↑ Piglet’s birth weight (kg)	< 0.01
Piglet mortality 24h after birth	↓ Piglet’s birth weight (kg)	< 0.01
	↑Cumulative birth interval (min)	0.01
Piglet mortality between 24h post-farrowing and weaning	↓ Colostrum intake (kg)	< 0.01
Stillbirth occurrence (yes/no)	↑Sows’ parity	0.02
	↑Cumulative birth interval (min)	< 0.01
Sow level		
Farrowing Duration (min)	↑Parity	0.02
	↑Stillborn piglets (n)	0.01
	↑Mummified piglets (n)	0.03
Average colostrum intake (g)	↓Sow’s rectal temperature 24h after farrowing (ºC)	< 0.01
	↓Live born piglets (n)	< 0.01

The up arrow (↑) indicates a positive association between the dependent and independent variables, and the down arrow (↓) indicates a negative association between the dependent and independent variables

**Fig. 1:**
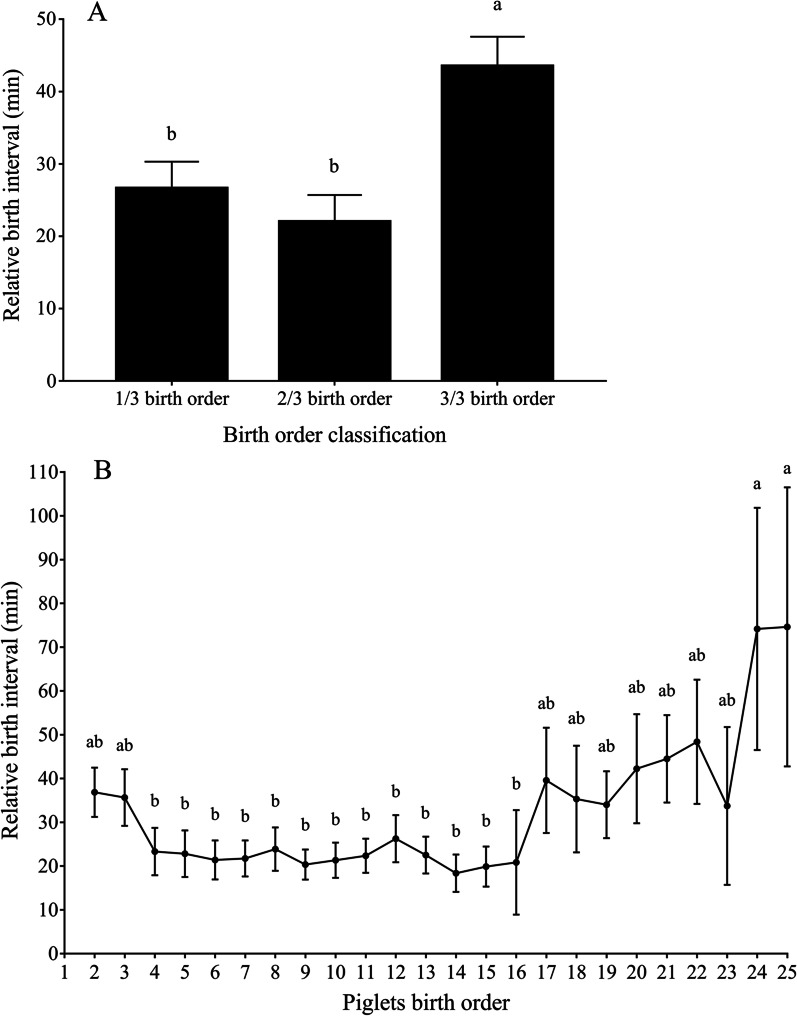
Inter-piglets birth interval (min) according to birth order. **A** Inter-piglet birth interval according to birth order grouped by terciles. Birth order between 2 and 7 was considered as the first tercile (1/3; n = 464 piglets) of piglets born, birth order between 8 and 16 was considered the second tercile (2/3; n = 430 piglets) and birth order between 17 and 25 was considered the last tercile (3/3; n = 279 piglets)). **B** Inter-piglet birth interval for each birth order. The birth interval of the first piglet (birth order 1) was not included as it was considered as zero. Data are presented as mean ± SEM. Different letter means statistical significance at *p* < 0.01

Piglets born in the last tercile had higher (*p* < 0.01) IPBI compared to piglets born in the first and second tercile (26.5 ± 3.8 min, 21.9 ± 3.8 min and 43.4 ± 4.17 min for first, second and last tercile, respectively) as shown in Fig. 1A. Additionally, the highest IPBI was observed in birth order 24 (75.1 min ± 11.1) and 25 (80 min ± 13.6). The IPBI of the birth order 24 and 25 was higher (*p* < 0.05) than the IPBI of the birth order 4 to 16, but it did not differ from the IPBI of the birth order 2, 3 and 17 to 23 (Fig. 1B).

The multivariable regression model considering IPBI as dependent variable is summarized in Tables 3 and 4. Both cumulative birth interval and piglet’s birth weight were positively associated (*p* < 0.01 and *p* = 0.03, respectively) with IPBI. It was estimated that an increase of one minute in cumulative birth interval increases 0.08 ± 0.01 min in IPBI and an increase of one kilogram in piglet’s birth weight increases IPBI in 6.25 ± 4.0 min (e.g., each 100 g of piglet’s birth weight increases IPBI in 37.5 s). In a multivariable model considering binary variables as independent variables affecting IPBI, the occurrence of both stillborn and manual intervention were also positively associated (*p* < 0.01) with IPBI, increasing it in 18.68 ± 3.26 and 98.03 ± 5.71 min, respectively.

**Table 3:** Multivariable model of cumulative birth interval and piglets birth weight on inter-piglet birth interval

Variable	Inter-piglet birth interval (min)*				Adjusted R^2^
	Estimate	SEM	95% confidence interval		
			Lower limit	Upper limit	
Intercept	1.95	5.11	−8.37	12.16	
Cumulative birth interval (min)	0.08	0.01	0.06	0.10	0.14
Piglet’s birth weight (kg)	6.25	4.00	−1.16	14.17	

This analysis was performed with 1188 piglets born from 58 sows

^*^The dependent variable was transformed to fit the normal distribution. The non-transformed data are presented in this table

Sow was considered as random variable in this statistical model

**Table 4:** Multivariable model of stillborn piglet and manual intervention on inter-piglet birth interval

Variable	Inter-piglet birth interval (min)				Adjusted R^2^
	Estimate	SEM	Confidence interval		
			Lower limit	Upper limit	
Intercept	19.99	2.24	15.59	24.43	0.34
Stillborn piglet (yes/no)	18.68	3.26	12.29	25.10	
Manual intervention (yes/no)	98.03	5.71	86.61	109.71	

This analysis was performed with 1188 piglets born from 58 sows

Sow was considered as random variable in this statistical model

**Fig. 2:**
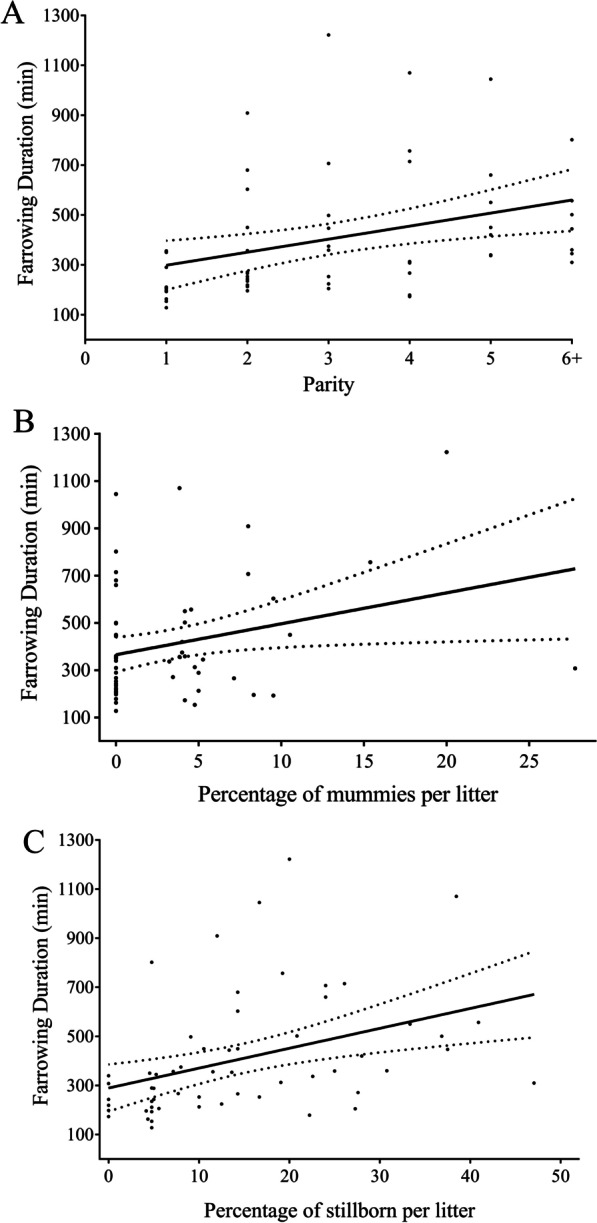
Univariable regression models showing the association between farrowing duration and percentage of mummified piglets per litter (**A**), parity (**B**) and percentage of stillborn per litter (**C**)

The multivariable regression model that evaluated variables affecting farrowing duration showed parity, number of stillborn piglets and number of mummified piglets as significant (*p* = 0.02, *p* = 0.01 and *p* = 0.03, respectively) variables (Fig. 2). It was estimated that each mummified or stillborn piglet increased the duration of farrowing with 67.08 ± 25.65 and 27.98 ± 11.98 min, respectively (Table 5). An increase of one unit in parity was associated with an increase of 28.91 ± 18.75 min in farrowing duration.

**Table 5:** Multivariable model of parity, stillborn piglets and mummified piglets on farrowing duration

Variable	Farrowing Duration (min)*				Adjusted R^2^
	Estimate	SEM	Confidence interval		
			Lower limit	Upper limit	
Intercept	183.41	60.76	61.58	305.23	0.35
Parity	28.91	18.75	−8.67	66.49	
Stillborn piglets (n)	27.98	11.98	3.96	52.00	
Mummified piglets (n)	67.08	25.65	15.65	118.50	

This analysis was performed with 58 sows

*The dependent variable was transformed to fit the normal distribution. The non-transformed data are presented in this table

The multivariable regression model considering average colostrum intake by piglet per sow as dependent variable is summarized in Table 6. Both sow’s rectal temperature 24h after farrowing and the number of liveborn piglets were negatively associated (*p* < 0.01) with average colostrum intake per piglet. Each extra liveborn piglet was associated with an average decrease of 13.3 ± 2.1 g of colostrum per piglet, and an increase in 0.1ºC in the sow’s rectal temperature was associated with an average decrease of 79.1 ± 24.5 g of colostrum per piglet. Birth weight was positively associated (*p* < 0.01) with individual colostrum intake per piglet (Table 7). Thus, at a piglet level, an increase of one kg in a piglet’s birth weight is associated with an increase of 0.388 ± 0.012 kg in individual colostrum intake.

**Table 6:** Multivariable model of sow’s rectal temperature 24h after farrowing and number of live born piglets on average colostrum intake by piglets

Variable	Average colostrum intake (g)				Adjusted R^2^
	Estimate	SEM	Confidence interval		
			Lower limit	Upper limit	
Intercept	3526.7	925.8	1671.3	5382.2	0.52
Sow’s rectal temperature 24h after farrowing (ºC)	−79.1	24.5	−128.3	−30.0	
Live born piglets (n)	−13.3	2.1	−17.4	−9.1	

This analysis was performed with 58 sows

**Table 7:** Univariable model of piglet’s birth weight on individual colostrum intake

Variable	Colostrum intake (g)				Adjusted R^2^
	Estimate	SEM	Confidence interval		
			Lower limit	Upper limit	
Intercept	−144.9	16.69	−177.2	−111.7	0.66
Piglet’s birth weight (kg)	388.6	12.35	364.3	412.8	

This analysis was performed with 908 piglets born from 58 sows. Stillborn, mummified piglets and piglets that died prior to 24h after birth were not included

**Fig. 3:**
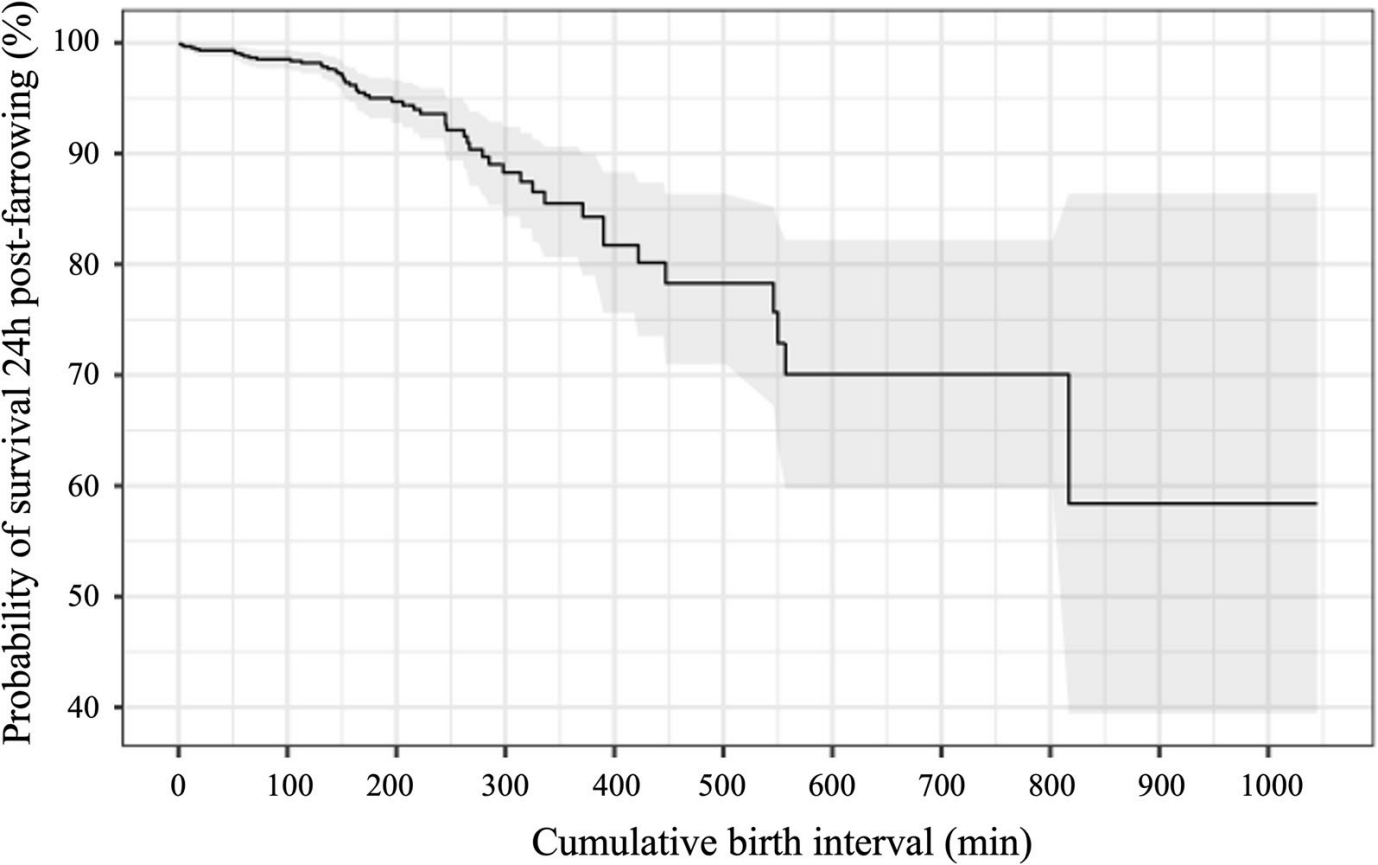
Probability of piglets being alive until 24h post-farrowing according to their respective cumulative birth interval. 983 piglets were included in this analysis. Stillborn and mummified piglets were not included

The probability of survival 24h post-farrowing decreased as the cumulative birth interval increased (Fig. 3). Similarly, Piglet’s mortality 24h post-farrowing was significant associated (*p* < 0.01) with piglet’s birth weight and cumulative birth interval, as shown in Table 8. Piglet’s birth weight and mortality 24h after birth were negatively associated, while cumulative birth interval was associated positively with piglet mortality 24h post-farrowing. Furthermore, only colostrum intake was found as a significant (*p* < 0.01) variable associated with piglet mortality from 24h post-farrowing until weaning (Table 9).

**Table 8:** Multivariable model of piglet’s birth weight and cumulative birth interval on piglet mortality 24h after birth

Variable	Piglet mortality 24h after birth				Adjusted R^2^
	Estimate	SEM	Confidence interval		
			Lower limit	Upper limit	
Intercept	−0.85	0.63	−2.11	0.36	0.32
Piglet’s birth weight (kg)	−2.64	0.57	−3.80	−1.55	
Cumulative birth interval (min)	0.003	0.001	0.001	0.004	

This analysis was performed with 983 piglets born from 58 sows. Stillborn and mummified piglets were not included

Sow was considered as a random variable in this statistical model

**Table 9:** Final multivariable model showing the effect of colostrum intake on piglet mortality between 24h post-farrowing and weaning

Variable	Piglet mortality between 24h post-farrowing and weaning				
	Estimate	SEM	Confidence interval		Adjusted R^2^
			Lower limit	Upper limit	
Intercept	1.22	0.22	0.78	1.69	0.48
Colostrum intake(g)	−0.011	0.57	−0.013	−0.009	

This analysis was performed with 908 piglets born from 58 sows. Stillborn, mummified piglets and piglets that died prior to 24h after birth were not included

Sow was considered as random variable in this statistical model

**Fig. 4:**
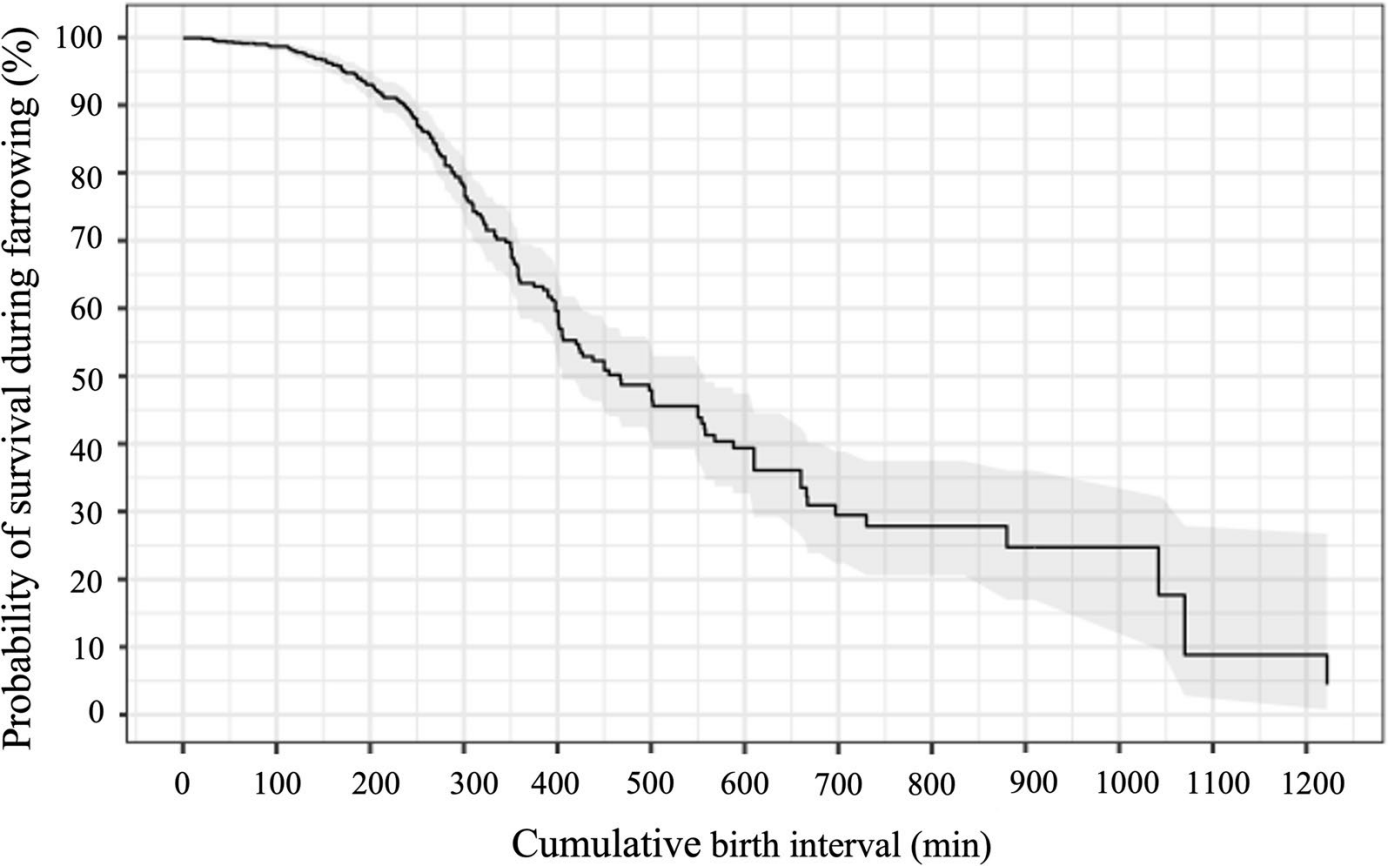
Probability of piglets being alive during farrowing according to the cumulative birth interval

The probability of piglet’s survival during farrowing decreased as the cumulative birth interval increased (Fig. 4). The multivariable regression model considering the occurrence (yes or no) of stillbirth as dependent variable is summarized in Table 10. Both sow’s parity and cumulative birth interval were positively associated (*p* = 0.02 and *p* < 0.01, respectively) with the presence of a stillborn piglet.

**Table 10:** Multivariable model of Sow’s parity and cumulative birth interval on stillbirth occurrence. 1152 piglets born from 58 sows were included in this analysis. Mummified piglets were not included

Variable	Stillbirth occurrence (yes/no)				
	Estimate	SEM	Confidence interval		Adjusted R^2^
			Lower limit	Upper limit	
Intercept	−3.30	0.26	−3.87	2.80	0.26
Sow’s parity	0.12	0.05	0.02	0.22	
Cumulative birth interval (min)	0.004	0.001	0.003	0.006	

This analysis was performed with 1152 piglets born from 58 sows. Mummified piglets were not included

Sow was considered as a random variable in this statistical model

Only piglets born alive (0) and stillborn piglets (1) were included in this analysis (1152 piglets). Mummified piglets were excluded.

## Discussion

In the present study, piglets born in the last tercile of birth order had higher IPBI compared to piglets born in the first and second terciles. This result agrees with another result from the present study where a positive linear association was found between cumulative birth interval and IPBI. These results together allow the interpretation that as the farrowing progresses, the piglet-piglet interval increases and, hence, the last piglets born are more prone to suffer from the detrimental effects of hypoxia. Van Dijk et al. [22] found a curvilinear association between IPBI and birth order evidencing that an increase in IPBI in the last piglets born occurred in five different breeds. Uddin et al. [8] also found that the last quartile of piglets born (birth order from 16 to 20) had the highest IPBI compared to piglets born earlier. Contrastingly, van Rens and van der Lende [23] found that the IPBI decreased linearly according to birth order. However, the average number of piglets was considerably lower in the latter study (10.1 total born piglets) compared to the present study (20.6 total born piglets).

It is estimated that sows’ energy requirement on the day of farrowing is 1.6 times higher compared to late gestation, mainly because of colostrum production, physical activity related to nest-building behavior, and the labor during farrowing [24]. Collectively, the results from the present study suggest that as farrowing proceeds, there is a depletion of energy reserves and sows get exhausted. Consequently, farrowing may be impaired by inadequate energy availability for uterine contractions [25, 26], which leads to higher IPBI towards the end of farrowing and a greater risk of low viability of the piglets born last. However, more studies are needed to confirm the hypothesis of energy depletion towards the end of farrowing as it was not tested in the present study. Also, it can be assumed that, given the longer farrowing durations, modern hyperprolific sows are more susceptible to exhaustion during farrowing than the older and less prolific genotypes. Although some traits related to piglets have been considered in this study, sow-related variables were found as the main factors increasing IPBI and farrowing duration. More studies are needed to better understand the effects of piglet-related traits on farrowing kinetics.

Piglet’s birth weight was also associated with longer IPBI. This finding has already been demonstrated in Large White x Meishan [22] and in Dutch Landrace litters [23]. Van Rens and van der Lende [23] showed that not the weight of the piglets but the thickness of the placenta is responsible for increased birth intervals. The positive association between the piglet’s birth weight and IPBI found in the present study might not have an important effect on the piglet’s viability as it was shown a minor increase of only 37.5 s for each 100 g increase in the piglet’s birth weight. Additionally, heavier piglets seem to be more resistant to intrapartum asphyxia than lighter piglets [7, 27]. Thus, the benefits of increased piglet birth weight might overcome its potential negative effect on IPBI.

The positive association between parity and farrowing duration found in the present study agrees with other studies [16, 28]. It is generally accepted that older sows are more susceptible to prolonged farrowing and high stillbirth rate, possibly due to poor muscle contractions of the uterus [29, 30]. Therefore, particular attention should be paid to older sows with evidence of dystocia (e.g., prolonged farrowing, high IPBI, meconium-stained piglets, lack of uterine contractions), to properly apply interventions accelerating the farrowing process, such as udder stimulation, postural changes, injection of exogenous uterotonics or manual intervention.

The present study showed a positive association between stillborn piglets and farrowing kinetics, which agrees with several other studies [25, 31, 32]. It has already been shown that the occurrence of stillborn piglets is higher towards the end of farrowing especially in hyperprolific sows [8, 9]. Then, both cumulative interval and IPBI increase the risk of stillborn piglets and one can potentiate the effect of the other. The presence of a mummified piglet was also positively associated with farrowing duration. It is argued that live born piglets can actively move through the pelvic canal and, their physical movements may stimulate further uterine contractions [33]. The size of the piglet may also exert some influence on the sow’s capacity for uterine and abdominal contraction since the estimate of the increase in farrowing duration associated with mummified piglets in the current study was 2.4-fold higher than the increase associated with stillborn piglets, as mummified piglets are normally smaller and lighter. Therefore, obstetric interventions (e.g. injection of uterotonics) may be used in the last tercile of farrowing to sows that present a duration of farrowing ≥ 300 min without birth canal obstruction [34]. In case of a high stillbirth rate or high mummification occurrence in early birth orders (more than ≥ 2 piglets prior to the 15^th^ piglet born), interventions may be considered even in the beginning or middle of the farrowing to avoid the cumulative effect of IPBI and cumulative farrowing duration on the risk of stillbirth occurrence.

Although a cumulative effect of IPBI and farrowing duration may occur, the effect of farrowing duration seems to be more deleterious to piglets’ survivability since cumulative birth interval and not IPBI was associated with stillbirth occurrence and piglets’ mortality 24h after farrowing. In agreement, Langendijk et al. [7] showed that the risk of stillbirth only increases significantly when IPBI exceeds 90 min, whereas the duration of farrowing increased the risk of stillbirth cumulatively with every 2 h. This also explains why the stillbirth rate and piglets’ vitality are impaired as birth order increases; piglets born later are more affected by uterine contractions, which impairs their blood supply leading to anaerobic metabolism, asphyxia and, possibly, brain damage [9]. Therefore, decreasing farrowing duration is crucial to minimize piglets’ losses during and after farrowing.

Neonatal mortality is a major cause of pre-weaning losses and the first 24h after birth are the most critical period [35], accounting for 28% of preweaning mortality [36]. The present study showed that piglet mortality 24h after farrowing is negatively associated to the piglet’s birth weight. This result was consistently reported previously [37–40]. These studies had on average 9.5, 12.5, 12.1 and 13.3 total piglets born, which means that for more prolific genotypes as used in the present study (average of 20.6 total piglets born), this result might be even more relevant as they have a higher occurrence of light born piglets. Low birthweight piglets are more at risk due to low energy reserves and a poor ability to compete at the udder [41]. Ferrari et al. [40] demonstrated that the highest neonatal mortality occurred in piglets with birth weight < 1.200 kg and associated this fact with an impaired colostrum intake (< 250 mL). In agreement, Declerck et al. [42] demonstrated that colostrum intake was positively associated with weaning weight and negatively associated with preweaning mortality. The present study also showed that lower birth weight is associated with lower colostrum intake which agrees with other studies [40–43]. Therefore, remarkable attention and care must be designated to the lighter piglets within a litter to increase their colostrum intake and minimize piglet mortality.

Impaired piglet’s colostrum intake may also be attributed to sow-related factors [43, 44]. Although the average colostrum intake may be affected by the global vitality of the litter, it is more frequently associated with the capacity of the sow to produce enough colostrum for the whole litter [42]. Hasan et al. [45] and Declerck et al. [42] estimated a decrease of 9.4 g and 9.0 g for each extra live born piglet, respectively, while the present study estimated a decrease of 13.3 g of average piglet’s colostrum intake by each extra live born piglet. This represents 40% (3.9 g) less average colostrum intake for each extra live born in comparison to the abovementioned studies. This difference may be associated with the greater prolificacy of the sows included in the present study and corroborates with the assumption that large litters are more prone to insufficient colostrum intake [44, 46].

The average piglet’s colostrum intake was also negatively associated with the sow’s rectal temperature 24 h after farrowing. An increase in rectal temperature is the earliest clinical sign to predict exacerbated inflammatory response during peripartum in sows [47, 48]. It can be hypothesized that disturbances of homeostasis caused by an increased inflammatory state and detected by increased rectal temperature can be detrimental to colostrum yield. However, the current knowledge in the literature cannot fully explain this association and the data presented in this study is not enough to draw a firm conclusion on this topic.

## Conclusion

The time elapsed from the onset of farrowing (expulsion of the first piglet) until the birth of a given piglet (cumulative birth interval) emerged as one of the most important factors influencing piglet survivability during and 24h post farrowing in hyperprolific genotypes. Although less relevant to piglets’ survivability, the IPBI may also increase the stillbirth rate, especially when acting together with cumulative birth interval. Therefore, strategies to decrease the farrowing duration of modern sows without impairing piglet’s vitality and maternal health must be developed. Moreover, older sows should be more carefully monitored during farrowing and obstetric interventions must be considered in sows showing evidence of dystocia.

## Materials and methods

The study protocol was approved by the Ethical Committee of the Faculty of Veterinary Medicine and the Faculty of Bioscience Engineering, Ghent University (EC2019-26), as well as by the Flemish governmental agency for animal welfare (DWZ/ER/20/1.15/).

### Farm and herd description

The study was performed in a commercial farrow-to-finish farm with an average herd size of 500 DanBred sows (Landrace x Yorkshire) and practicing a 4-week batch production system. Piglets were weaned at 21 days of age. Sows and gilts were transferred to the farrowing unit three to seven days before the expected farrowing date where they were housed in conventional farrowing crates until weaning. Prior to moving them to the farrowing unit, the animals were fed a gestation diet. After arrival in the farrowing unit, they received a transition diet until two to three days after the last sow had farrowed. From then onwards until weaning, sows were fed with lactation diet. The precise composition of the different feeds can be found in Schoos et al. [49]. All the animals had ad libitum access to drinking water via a drinking nipple. The temperature in the farrowing unit varied between 24.5 ºC and 25.0 ºC throughout the study.

Sows and gilts that farrowed prior to gestation day 115 and after day 117 (considering the first insemination day as day 0 of pregnancy) were not considered for the study. Induction of farrowing was not applied. Cross-fostering and split suckling were allowed only after 24h after the onset of the farrowing.

### Measurements and calculations

The backfat thickness was considered as the average of measurements performed in the left and right side of the sows at the P2 position (Renco Lean-Meater, MN, USA) at the entrance in the farrowing room, farrowing day, and weaning. The rectal temperature was daily assessed between 9 and 11 a.m. from the day of entrance in the farrowing unit until seven days after the last sow had farrowed.

Total born, live born, stillborn and mummified piglets were recorded. The average birth interval was calculated by dividing the farrowing duration by the number of total born piglets in the litter. The IPBI was calculated as the time elapsed between the birth of a piglet and the birth of the next piglet. The cumulative interval was calculated as the time elapsed between the onset of farrowing (expulsion of the first piglet) and the birth of a piglet.

At birth, piglets were weighed and received an individual ear tag, and 24h (23–25 h) later, the piglets were weighed again to estimate colostrum intake and colostrum yield. Colostrum intake was estimated based on the mechanistic model as described by Theil et al. [50]. If the mathematical model indicated a negative value, the colostrum intake was considered zero. The colostrum yield of each sow was calculated as the sum of the individual piglets’ colostrum intake within a litter. Potential colostrum intake of piglets dying within 24h after birth was not considered. Piglets’ mortality was recorded daily throughout the lactation. Piglets were weighed at weaning and average litter gain was calculated by the difference of birth weight and weaning weight divided by the number of days in lactation.

### Statistical analyses

The assumption of normality and homogeneity of variances were graphically evaluated (histogram, normal probability plot of residuals) and tested by Shapiro–Wilk and Barlett, respectively. When needed, dependent variables were transformed in order to meet the assumptions of the statistical model used. The data were presented as mean ± SEM and the results were considered significant at *p* < 0.05. Statistical analyses were performed using software R (R Core Team, version 4.2.0).

The birth order was categorized into three groups to analyze the IPBI according to the piglet’s expulsion, with 25 being the maximum birth order as the number of piglets from birth order 26 onwards was too low to fit the model adequately. The number of piglets born in each birth order is in Additional File 1: Table S1. For this, a birth order between 2 and 7 was considered as the first tercile (1/3) of piglets born, a birth order between 8 and 16 was considered the second tercile, and a birth order between 17 and 25 was considered the last tercile. If a sow had less than 17 piglets, it was considered only for the first and second tercile. The overall IPBI, as well as the IPBI in each of these three groups, were compared using a linear mixed model where the sow was considered as a random variable.

Univariable models were used to investigate the association between predicted and predictor variables, where each explanatory variable was included as a single fixed effect. Numerical and categorical independent variables with p ≤ 0.20 for the F-test in the simple model were selected and subjected to Pearson’s and Spearman’s correlation analysis to avoid multicollinearity between continuous variables and confounding problems between categorical variables. Based on the results from the univariable models, all factors with p ≤ 0.20 were included as independent variables in a multivariable analysis. After a stepwise elimination procedure, only independent variables with *p* < 0.05 were included in the final model. The elimination of independent variables in the stepwise procedure was performed according to the *p*-value; independent variables with higher *p*-values were eliminated earlier. The complete linear regression models, including inclusion and exclusion criteria and stepwise procedure are shown in Additional File 1: Table S2–S9.

Statistical models that had the dependent variable as a binary variable (piglets’ mortality before 24h post-farrowing, piglets’ mortality between 24h post-farrowing and weaning, and the occurrence of stillbirth) were analyzed by generalized linear mixed models fitted by binomial distribution.

Sow was considered as a random variable in statistical models that analyzed dependent variables at the piglet level (IPBI, piglets’ mortality until 24h post-farrowing, piglets’ mortality between 24h post-farrowing and weaning, and the occurrence of stillbirth).

Interaction between the variables included in the final model was tested and found to be non-significant for all models.

Survival analysis (Kaplan–Meier estimate) was performed using the “survival” package and “survfit” function with a confidence interval of 95%.

### Supplementary Information

**Additional file 1.** Supplementary Tables.

## Data Availability

The dataset used and/or analyzed during the current study is available from the corresponding author on request.
