# Supplementary material for: Relationship between piglets’ survivability and farrowing kinetics in hyper-prolific sows
Source: Porcine Health Manag. 2023 Aug 28;9:37. doi: 10.1186/s40813-023-00332-y (PMC10464185; doi:10.1186/s40813-023-00332-y)
Supplement: Supplementary file 1 — Additional file 1. Supplementary Tables. [file 40813_2023_332_MOESM1_ESM.docx]

**Supplementary Table S1, Additional File 1**: Number of piglets included in the inter-piglet birth interval analysis according to the birth order.

| Birth order | Number of piglets included in the analysis |
| --- | --- |
| 1 | 58 |
| 2 | 58 |
| 3 | 58 |
| 4 | 58 |
| 5 | 58 |
| 6 | 58 |
| 7 | 58 |
| 8 | 58 |
| 9 | 58 |
| 10 | 57 |
| 11 | 55 |
| 12 | 55 |
| 13 | 53 |
| 14 | 53 |
| 15 | 50 |
| 16 | 49 |
| 17 | 48 |
| 18 | 46 |
| 19 | 42 |
| 20 | 39 |
| 21 | 34 |
| 22 | 23 |
| 23 | 19 |
| 24 | 17 |
| 25 | 11 |

**Supplementary Table S2, Additional File 1**. The complete linear regression model, including inclusion and exclusion criteria, for the inter-piglet birth interval as dependent variable (relative to Table 3 in the manuscript)

| Variables | | *p-value* | Inclusion in the multivariate analysis | |  |
| --- | --- | --- | --- | --- | --- |
| Univariable analysis | | | | |  |
| Cumulative birth interval | | <.0001 | Included | |  |
| Colostrum intake | | 0.02 | Included | |  |
| Colostrum intake / body weight | | 0.82 | Excluded | |  |
| Body weight at birth | | <.0001 | Included | |  |
| Weight gain 24h after farrowing | | 0.22 | Excluded | |  |
| Average daily weight gain from birth to weaning | | 0.004 | Included | |  |
| Variables | | Final model  *p-value* | | Stepwise 2  *p-value* | Stepwise 1  *p-value* |
| Multivariable analysis with stepwise elimination | | | | | |
| Cumulative birth interval | | < 0.01 | | < 0.01 | < 0.01 |
| Colostrum intake | | - | | - | 0.70 |
| Body weight at birth | | 0.03 | | 0.02 | 0.05 |
| Average daily weight gain from birth to weaning | | - | | 0.80 | 0.70 |
|  | |  | |  |  |

**Supplementary Table S3**, **Additional File 1**. The complete linear regression model, including inclusion and exclusion criteria, for the inter-piglet birth interval as a dependent variable and binary variables as independent variables (relative to Table 4 in the manuscript)

| Variables | *p-value* | Inclusion in the multivariate analysis |
| --- | --- | --- |
| Univariable analysis | | |
| Stillborn piglets (yes / no) | < 0.01 | Included |
| Mummified piglets (yes / no) | 0.39 | Excluded |
| Manual intervention (yes / no) | < 0.01 | Included |
| Variables | Final model  *p-value* | |
| Multivariable analysis with stepwise elimination | | |
| Stillborn piglets (yes / no) | < 0.01 | |
| Manual intervention (yes / no) | < 0.01 | |

**Supplementary Table S4**, **Additional File 1**. The complete linear regression model, including inclusion and exclusion criteria, for the farrowing duration as dependent variable (relative to Table 5 in the manuscript)

| Variables | | *p-value* | Inclusion in the multivariate analysis | |
| --- | --- | --- | --- | --- |
| Univariable analysis | | | | |
| Rectal temperature at day of farrowing | | 0.21 | Excluded | |
| Rectal temperature 24h after farrowing | | 0.73 | Excluded | |
| Rectal temperature 48h after farrowing | | 0.07 | Included | |
| Parity | | < 0.01 | Included | |
| Gestation length | | 0.90 | Excluded | |
| Total piglets born | | 0.08 | Included | |
| Stillborn piglets | | < 0.01 | Included | |
| Mummified piglets | | 0.04 | Included | |
| Backfat at entrance in the farrowing room | | 0.60 | Excluded | |
| Backfat at farrowing | | 0.90 | Excluded | |
| Backfat at weaning | | 0.71 | Excluded | |
| Live born piglets | | 0.25 | Excluded | |
| Colostrum yield | | 0.57 | Excluded | |
| IgG concentration in colostrum | | 0.26 | Excluded | |
| Variables | Final model  *p-value* | | Stepwise 2  *p-value* | Stepwise 1  *p-value* |
| Multivariable analysis with stepwise elimination | | | | |
| Parity | 0.02 | | 0.02 | 0.04 |
| Stillborn piglets | 0.01 | | 0.02 | 0.02 |
| Mummified piglets | 0.03 | | 0.04 | 0.06 |
| Total piglets born | - | | 0.98 | 0.91 |
| Rectal temperature 48hr after farrowing | - | | - | 0.61 |

**Supplementary Table S5**, **Additional File 1**. The complete linear regression model, including inclusion and exclusion criteria, for the average colostrum intake by piglets per sow as a dependent variable (relative to Table 6 in the manuscript)

| Variables | *p-value* | | | Inclusion in the multivariate analysis | | | | | | |  |
| --- | --- | --- | --- | --- | --- | --- | --- | --- | --- | --- | --- |
| Univariable analysis | | | | | | | | | | |  |
| Rectal temperature on day of farrowing | 0.81 | | | Excluded | | | | | | |  |
| Rectal temperature 24h after farrowing | <0.01 | | | Included | | | | | | |  |
| Rectal temperature 48h after farrowing | 0.21 | | | Excluded | | | | | | |  |
| Parity | 0.36 | | | Excluded | | | | | | |  |
| Gestation length | 0.27 | | | Excluded | | | | | | |  |
| Live born piglets | < 0.01 | | | Included | | | | | | |  |
| Stillborn piglets | 0.57 | | | Excluded | | | | | | |  |
| Mummified piglets | 0.41 | | | Excluded | | | | | | |  |
| Backfat at entrance in farrowing room | 0.05 | | | Included | | | | | | |  |
| Backfat at farrowing | 0.07 | | | Included | | | | | | |  |
| Backfat at weaning | 0.23 | | | Excluded | | | | | | |  |
| Average litter weight gain from birth until weaning (g) | 0.08 | | | Included | | | | | | |  |
| IgG concentration in colostrum | 0.02 | | | Included | | | | | | |  |
| Variables | Final model  *p-value* | | Stepwise 4  *p-value* | | | Stepwise 3  *p-value* | | Stepwise 2  *p-value* | | Stepwise 1  *p-value* |  |
| Multivariable analysis with stepwise elimination | | | | | | | | | | |  |
| Rectal temperature 24h after farrowing | < 0.01 | < 0.01 | | | 0.01 | | 0.01 | | 0.02 | | |
| Live born piglets | < 0.01 | < 0.01 | | | < 0.01 | | < 0.01 | | < 0.01 | | |
| Backfat at entrance in farrowing room |  | - | | | - | | - | | 0.87 | | |
| Backfat at farrowing |  | 0.38 | | | 0.35 | | 0.36 | | 0.74 | | |
| IgG concentration in colostrum |  | - | | | - | | 0.56 | | 0.58 | | |
| Average litter weight gain from birth until weaning |  | - | | | 0.37 | | 0.43 | | 0.42 | | |

**Supplementary Table S6, Additional File 1**. The complete linear regression model, including inclusion and exclusion criteria, for the individual colostrum intake by piglet as a dependent variable (relative to Table 7 in the manuscript).

| Variables | | | *p-value* | Inclusion in the multivariate analysis | |
| --- | --- | --- | --- | --- | --- |
| Univariable analysis | | | | | |
| Cumulative birth interval | | | 0.25 | Excluded | |
| Inter-piglet birth interval | | | < 0.01 | Included | |
| Piglet birth weight | | | < 0.01 | Included | |
| Variables | | Final model  *p-value* | | Stepwise 2  *p-value* | |
| Multivariable analysis with stepwise elimination | | | | | |
| Relative birth interval | - | | | | 0.53 |
| Piglet birth weight | < 0.01 | | | | < 0.01 |

**Supplementary Table S7**, **Additional File 1** The complete linear regression model, including inclusion and exclusion criteria, for piglet’s mortality until 24h post-farrowing as dependent variable (relative to Table 8 in the manuscript)

| Variables | *p-value* | Inclusion in the multivariate analysis | | | | | |
| --- | --- | --- | --- | --- | --- | --- | --- |
| Univariable analysis | | | | | | | |
| Parity | 0.60 | Excluded | | | | | |
| Backfat at farrowing | 0.10 | Included | | | | | |
| Rectal temperature at farrowing | 0.11 | Included | | | | | |
| Rectal temperature 24h after farrowing | 0.19 | Included | | | | | |
| Total piglets born | 0.01 | Included | | | | | |
| Live born piglets | 0.05 | Included | | | | | |
| Average colostrum IgG concentration | 0.04 | Included | | | | | |
| Piglet birth weight | < 0.01 | Included | | | | | |
| Piglet’s birth order | 0.002 | Included | | | | | |
| Cumulative birth interval | 0.03 | Included | | | | | |
| Relative birth interval | 0.20 | Included | | | | | |
| Variables | Final model  *p-value* | | Stepwise 5  *p-value* | Stepwise 4  *p-value* | Stepwise 3  *p-value* | Stepwise 2  *p-value* | Stepwise 1  *p-value* |
| Multivariable analysis with stepwise elimination | | | | | | | |
| Rectal temperature at farrowing | - | | 0.19 | 0.16 | 0.07 | 0.07 | 0.06 |
| Rectal temperature 24h after farrowing | - | | - | - | 0.16 | 0.12 | 0.14 |
| Backfat at farrowing | - | | - | 0.42 | 0.60 | 0.60 | 0.60 |
| Live born piglets | - | | - | - | - | 0.87 | 0.57 |
| Piglet birth weight | < 0.01 | | < 0.01 | < 0.01 | < 0.01 | < 0.01 | < 0.01 |
| Cumulative birth interval | 0.01 | | 0.008 | 0.02 | 0.02 | 0.02 | 0.05 |
| Relative birth interval | - | | - | 0.43 | 0.40 | 0.41 | 0.42 |

**Supplementary Table S8**, **Additional File 1** The complete linear regression model, including inclusion and exclusion criteria, for the piglet’s mortality between 24h post-farrowing and weaning as the dependent variable (relative to Table 9 in the manuscript)

| Variables | *p-value* | Inclusion in the multivariate analysis | | | | |
| --- | --- | --- | --- | --- | --- | --- |
| Univariable analysis | | | | | | |
| Parity | 0.10 | Included | | | | |
| Backfat at farrowing | 0.15 | Included | | | | |
| Backfat at weaning | 0.84 | Excluded | | | | |
| Rectal temperature at farrowing | 0.56 | Excluded | | | | |
| Rectal temperature 24h after farrowing | 0.24 | Excluded | | | | |
| Rectal temperature 48h after farrowing | 0.01 | Included | | | | |
| Live born piglets | <.001 | Included | | | | |
| Individual colostrum intake | <.001 | Included | | | | |
| Average colostrum IgG concentration | 0.70 | Excluded | | | | |
| Piglet birth weight | <.001 | Included | | | | |
| Piglet’s birth order | 0.02 | Included | | | | |
| Cumulative birth interval | 0.40 | Excluded | | | | |
| Relative birth interval | 0.50 | Excluded | | | | |
| Variables | Final model  *p-value* | Stepwise 5  *p-value* | Stepwise 4  *p-value* | Stepwise 3  *p-value* | Stepwise 2  *p-value* | Stepwise 1  *p-value* |
| Multivariable analysis with stepwise elimination | | | | | | |
| Parity |  |  | - | - | - | 0.95 |
| Backfat at farrowing |  |  | - | - | - | 0.72 |
| Rectal temperature 48h after farrowing |  | 0.23 | 0.22 | 0.20 | 0.17 | 0.20 |
| Live born |  |  | - | - | 0.41 | 0.38 |
| Individual colostrum intake | <0.01 | <0.01 | <0.01 | <0.01 | <0.01 | <0.01 |
| Piglet’s birth weight |  |  | - | 0.44 | 0.39 | 0.25 |
| Piglet´s birth order |  |  | 0.25 | 0.26 | 0.20 | 0.31 |

| Variable | *p-value* | Inclusion in the multivariate analysis |
| --- | --- | --- |
| Univariate analysis | | |
| Sow´s Parity | < 0.01 | Included |
| Sow´s backfat at farrowing | 0.81 | Excluded |
| Cumulative birth interval | < 0.01 | Included |
| Relative birth interval | < 0.01 | Included |
| Variables | Final model  *p-value* | Stepwise 1  *p-value* |
| Multivariable analysis with stepwise elimination | | |
| Sow´s parity | 0.02 | 0.02 |
| Cumulative birth interval | < 0.01 | < 0.01 |
| Relative birth interval | - | 0.41 |

**Supplementary Table S9**, **Additional File 1** The complete linear regression model, including inclusion and exclusion criteria, for stillbirth occurrence (yes or no) as dependent variable (relative to Table 10 in the manuscript)
